# Chitosan Encapsulated Meloxicam Nanoparticles for Sustained Drug Delivery Applications: Preparation, Characterization, and Pharmacokinetics in Wistar Rats

**DOI:** 10.3390/molecules27217312

**Published:** 2022-10-27

**Authors:** Muralidhar Yegireddy, Prakash Nadoor, Suguna Rao, Pavithra Balekatte Hanumanthu, Rashmi Rajashekaraiah, Santhosh Chickankandahalli Ramachandrappa, Girish Mallikarjun Halemani, Sravanthi Mannem, Tollamadugu Naga Venkata Krishna Vara Prasad, Sunilchandra Ubaradka

**Affiliations:** 1 Department of Veterinary Pharmacology and Toxicology, Veterinary College, Hebbal, Bengaluru 560 024, Karnataka, India; 2 Karnataka Veterinary, Animal and Fisheries Sciences University, Bidar 585 401, Karnataka, India; 3 Veterinary College, Vinobanagar, Shivamogga 577 204, Karnataka, India; 4 Department of Veterinary Pathology, Veterinary College, Hebbal, Bengaluru 560 024, Karnataka, India; 5 Department of Veterinary Pharmacology and Toxicology, Veterinary College, Gadag 582 101, Karnataka, India; 6 Department of Veterinary Anatomy, Veterinary College, Hebbal, Bengaluru 560 024, Karnataka, India; 7 State Level Diagnostic Laboratory, Sri Venkateswara Veterinary University, Tirupati 517 502, Andhra Pradesh, India; 8 Nanotechnology Laboratory, Regional Agricultural Research Station, ANGRAU, Tirupati 517 502, Andhra Pradesh, India; 9 Department of Veterinary Pharmacology and Toxicology, Vinobanagar, Shivamogga 577 204, Karnataka, India

**Keywords:** meloxicam, chitosan, nanoparticles, pharmacokinetics, Wistar rats

## Abstract

Meloxicam (MLX) is currently used in the therapeutic management of both acute and chronic inflammatory disorders such as pain, injuries, osteoarthritis, and rheumatoid arthritis in both humans and animals. Gastrointestinal toxicity and occasional renal toxicity were observed in patients taking it for a long-term period. Meloxicam’s late attainment of peak plasma concentration results in a slow onset of action. The goal of the current study was to prepare and characterize chitosan encapsulated meloxicam nanoparticles (CEMNPs) with high bioavailability and less gastro intestinal toxicity in order to prevent such issues. The size of the prepared CEMNPs was approximately 110–220 nm with a zetapotential of +39.9 mV and polydispersity index of 0.268, suggesting that they were uniformly dispersed nanoparticles. The FTIR and UV-Vis spectroscopy have confirmed the presence of MLX in the prepared CEMNPs. The pharmacokinetics have been studied with three groups of male Wistar rats receiving either of the treatments, viz., 4 mg·kg^−1^ of MLX and 1 or 4 mg·kg^−1^ of CEMNPs. Plasma samples were collected until 48 h post administration, and concentrations of MLX were quantified by using reverse (C_18_) phase HPLC. Non-compartmental analysis was applied to determine pharmacokinetic variables. Upon oral administration, the maximum concentration (C_max_) was reached in 4 h for CEMNPs and 6 h for MLX. The mean area under the plasma MLX concentration-time curve from ‘zero’ to infinity (AUC_0–∞_), half-life (t_1/2β_), and mean resident time (MRT) of 1 mg·kg^−1^ of CEMNPs was 1.4-, 2-, and 1.8-fold greater than 4 mg·kg^−1^ of MLX. The prepared CEMNPs demonstrated quicker absorption and prolonged release along with a significant improvement in the bioavailability of MLX, paving a prospective path for the development of drugs with enhanced bioavailability with less side effects.

## 1. Introduction

The expression of pain consequent to inflammation is a necessity, and it favors veterinarians to identify and treat the cause of pain, which will inherently improve the overall welfare of the animal [1]. Part of treating the pain is understanding the mechanisms behind analgesic drugs in both their pharmacokinetic and pharmacodynamic properties. It is the information gathered through these investigations that helps to build a better understanding with which analgesic drugs will best treat a variety of painful experiences that may arise [2].

Non-steroidal anti-inflammatory drugs (NSAIDs), which are useful in alleviating pain, fever, and inflammation due to the release of prostaglandins, are among the most frequently prescribed medications in both human and veterinary medicine globally [3]. Overdose of NSAIDs and their chronic use are associated with adverse effects such as mucosal lesions, peptic/gastric ulcers, intestinal perforation, bleeding, hepatic, or renal damage with the potential increase in the risk of heart attack and stroke [4,5].

The NSAIDs act by inhibiting the action of the specific membrane-bound isomerase enzymes, i.e., cyclooxygenase 1 (COX-1) and cyclooxygenase 2 (COX-2). Many tissues produce constitutive prostaglandins, i.e., COX-1-related prostaglandins involved in the maintenance of a number of physiological processes including hemostasis, the protection of gastrointestinal mucosa, and renal blood flow. Contrarily, COX-2 primarily generates inducible prostaglandins, which are regarded as ‘non-physiologic’, and maintains features of inflammation including vasodilatation, changes in capillary permeability, potentiation of other chemical mediators of inflammation, chemotaxis, and hyperalgesia [6,7]. The adverse effects associated with NSAIDs are mainly related to the gastrointestinal tract, as these drugs non-selectively inhibit both COX enzymes. The loss of gastrointestinal protective mechanisms results from the inhibition of COX-1 constitutive prostaglandins that regulate blood flow to the gastric mucosa and stimulate bicarbonate and mucus production. This disrupts the alkaline protective barrier of the gut, allowing diffusion of gastric acid back into the mucosa, injuring cells and blood vessels, and causing gastritis and ulceration. The NSAIDs that inhibit COX-2 may be preferable because they prevent the production of COX-2 prostaglandins, which induce the clinical symptoms of inflammation, and because they have less of an impact on COX-1 prostaglandins, which have several homeostatic qualities [7].

Meloxicam (MLX) is an enolic acidic NSAID that has relative COX-2 selectivity. It is a commonly used NSAID in veterinary medicine for its anti-inflammatory, analgesic, and antipyretic activities [8,9,10,11]. Unlike other oxicam NSAIDs or other traditional COX inhibitors, MLX, with additional 5-methyl-2-thiazolyl moiety in the pyridine ring, facilitates preferential selectivity on the inhibition of COX-2 over COX-1 [8], but at higher doses it inhibits both [11]. MLX proved effective for treating rheumatoid arthritis, osteoarthritis, lumbago, and other excruciating situations such as injuries, cancer surgery, and dental infections [12]. MLX has recently been connected to nephrotoxicity, hepatotoxicity, and stomach metaplasia in a rat [13,14,15]. The aqueous thermodynamic solubility and dissolution rate of MLX is poor to low. However, MLX has an 89% bioavailability after dissolution [16], and its poor dissolving property restricts both its absorption rate and onset of action [17,18], which in turn effects the efficacy of MLX. The half-life of MLX is low compared to other NSAIDs [19,20,21,22]. There is need for the development of formulations with sustained release for attaining a rapid onset of action.

There is rising interest in the creation and optimization of drug-delivery systems due to the intricacy of some diseases and the intrinsic toxicity of some drugs. In order to reduce the unfavorable side effects of conventional treatments, a new branch of alternative medicine has been developed that more accurately targets the illness spot without damaging the healthy cells. Recently, the nanotechnology approach has been employed to improve the outcomes of both conventional and novel pharmaceutical illness therapies. Due to their tiny size, nanoparticles (NPs) are the most recent innovation in the field of medication delivery and are being used in a more targeted manner to reach target sites (1 to 100 nm) [23]. In several scientific domains, particularly in the pharmaceutical sector, nanotechnology provides significant benefits for the formulation of dosage forms. It is employed to increase the drug effectiveness, enhance dosage form stability, and provide less expensive dosage forms with fewer side effects, all of which promote patient compliance [24,25]. Additionally, the various nano dosages help to promote drug stabilization and absorption and facilitate the transit of poorly soluble medications into the cell because of their large surface area [25].

To increase drug bioavailability or precise targeted distribution at the site of action, polymeric nanoparticles stand out as a crucial instrument. Polymers have the potential to be suitable for meeting the criteria of each unique medication delivery system due to their versatility [26]. In general, polymeric nanoparticles can be optimized to improve the drug bioavailability, either by enhancing their solubility or enabling their transit through the biologic membranes to increase their absorption [27].

Chitosan, a naturally occurring hydrophilic cationic polysaccharide obtained from crustacean shells and fungal cell walls comprised of randomly dispersed β-1-4 linked D-glucosamine and N-acetylglucosamine, is one of the extensively used polymers for the synthesis of new drug delivery systems. Recently, the US FDA approved chitosan for the delivery of nanoparticles [28]. Due to its nontoxic nature, bio-adhesive property, biocompatibility, biodegradable property, and target specificity, chitosan can be used to prepare nanoparticles for drug delivery [29,30,31]. Chitosan-based nanomaterials exhibit superior drug absorption due to their GI luminal protection, mucoadhesive nature, permeability enhancement, controlled drug release, and efflux inhibition [30]. In recent times, few attempts have been made to synthesize MLX nanoparticles for effective delivery [17,18,32,33]. After a thorough review of the literature, and with the available information regarding chitosan-encapsulated meloxicam nanoparticles, the current investigation was carried out to explore the beneficial effects of chitosan-encapsulated meloxicam nanoparticles with respect to their pharmacokinetic behavior in male Wistar rats.

## 2. Results and Discussion

### 2.1. Preparation and Characterization of CEMNPs

In the present study, the CEMNPs were prepared by the ionic gelation crosslinking process. Due to the protonation of the amine group of chitosan in acidic pH, it interacts with polyanion TPP. The instantaneous formation of CEMNPs were observed upon adding the MLX dissolved in a methanol and TPP solution mixture to the chitosan solution. Because of the interaction between the negatively charged TPP and MLX, as MLX exists as an anion in neutral or weakly basic solution [34] with positively charged chitosan during ionic gelation, this resulted in the formation of CEMNPs.

**Figure 1:**
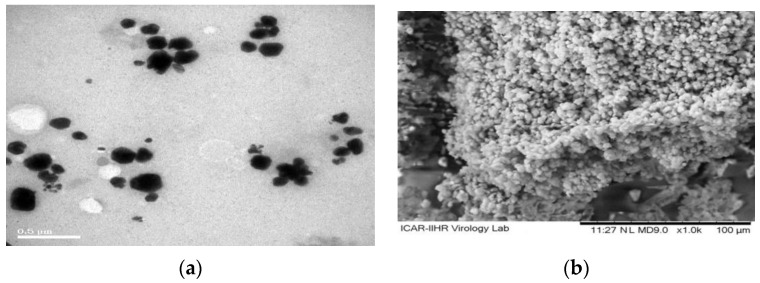
Transmission (**a**) and scanning electron microscopy (**b**) representative images of chitosan-encapsulated meloxicam nanoparticles.

**Figure 2:**
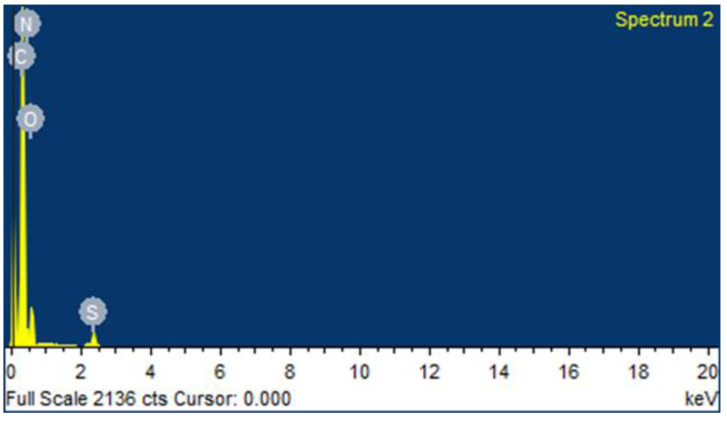
Energy dispersive spectroscopy (EDS) analysis of chitosan-encapsulated meloxicam nanoparticles (CEMNPs).

The transmission electron microscopic (TEM) and scanning electron microscopic (SEM) micrographs of the CEMNPs are shown in Figure 1a and Figure 1b, respectively. The electron microscopic analysis revealed that the shape of the CEMNPs was round to cuboidal in shape, with a size distribution of 110–200 nm. The energy-dispersive spectroscopy analysis (Figure 2 and Supplementary Table S1) revealed the presence of C, N, O, and S, which confirmed the presence of MLX in the prepared CEMNPs (Chitosan—C_12_H_24_N_2_O_9_; MLX—C_14_H_13_N_3_O_4_S_2_).

**Figure 3:**
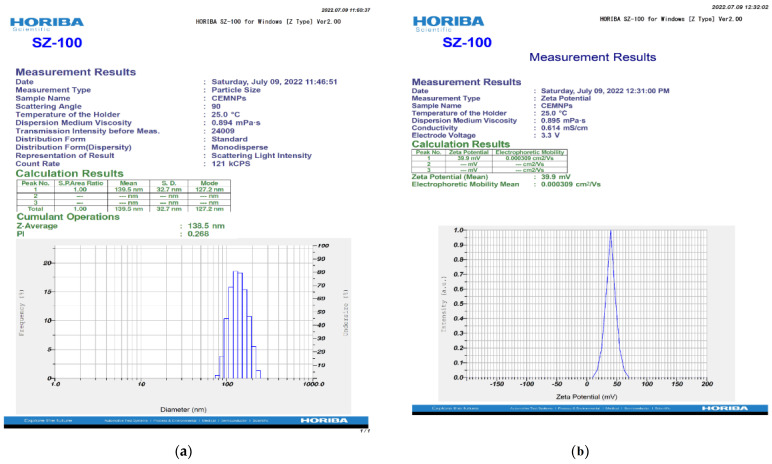
Hydrodynamic diameter (**a**) and *zeta* potential (**b**) of chitosan-encapsulated meloxicam nanoparticles (CEMNPs) in carboxymethyl cellulose (CMC; 0.1% *w*/*v*).

The DLS technique is considered a sensitive and valuable method for measurement of NPs. This method yields a particle’s hydrodynamic diameter [35]. The hydrodynamic diameter of the CEMNPs was measured in 0.1% carboxymethyl cellulose (CMC), which yielded 138.5 nm (Figure 3a). The previous studies with chitosan nanoparticles reported the size range of 90–690 nm [33,36,37,38].

The uniformity of particles in the solution was estimated using the polydispersity index value. A larger size distribution in the particle sample is indicated by higher polydispersity index values. The range of the polydispersity index is between ‘0’ and ‘1′, with ‘0’ denoting monodisperse and ‘1′ denoting polydisperse [39]. The polydispersity index of the CEMNPs measured in 0.1% CMC was 0.268 (Figure 3a), indicating that the prepared nanoparticles were monodispersed.

The *zeta* potential is a physical property exhibited by any particle in suspension. It depends on the surface charge and is important for the stability of NPs in suspension. According to conventional wisdom, charged particles with *zeta* potentials of above +30 mV or below −30 mV exhibit adequate electrostatic repulsion to provide colloidal stability [40]. In the present study, the prepared CEMNPs were positively charged with a *zeta* potential of +39.9 mV (Figure 3b). Due to the presence of free amine groups on the polymer, the positive *zeta* potential value of CEMNPs shows that the nanoparticle surface has positively charged [41,42]. In the previous studies, the *zeta* potential for chitosan nanoparticles was reported as positively charged [33,36,37,38].

**Figure 4:**
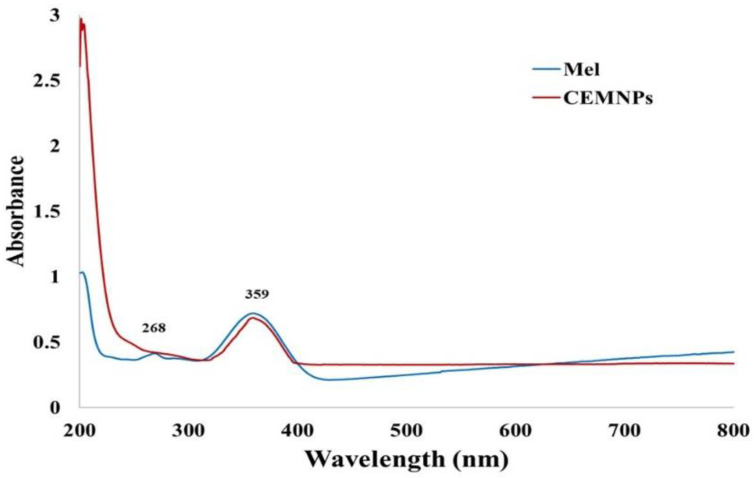
UV spectrum of meloxicam and chitosan-encapsulated meloxicam nanoparticles (CEMNPs).

The UV spectral analysis of MLX and the CEMNPs was obtained by carrying the scanning over the wavelength range of 200–400 nm, and the resulting spectrum is depicted in Figure 4. The primary peak was found to be around 359 nm. The prepared CEMNPs showed similar peaks with less intensity, which confirmed the presence of MLX. These results are in good agreement with the previous reports [43,44,45,46,47].

**Figure 5:**
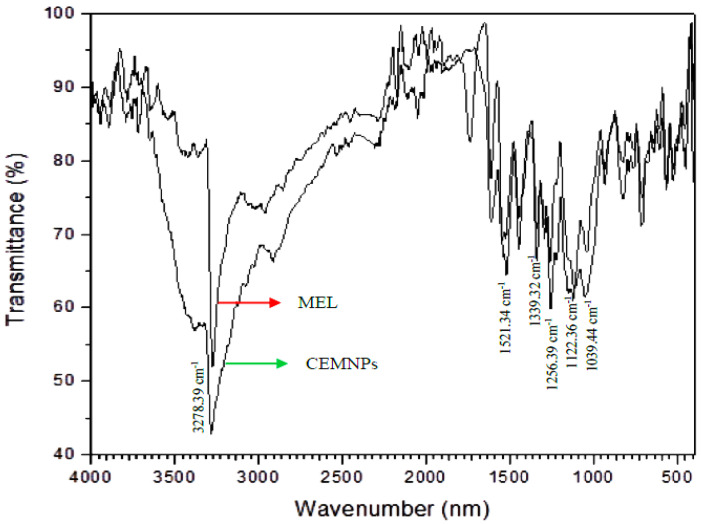
FTIR spectrum of meloxicam and chitosan-encapsulated meloxicam nanoparticles (CEMNPs).

The FT-IR spectrum of MLX and the CEMNPs was depicted in Figure 5. MLX showed a spectrum with characteristic peaks at 3278.39 cm^−1^ (N-H stretching vibrations of secondary amide), 1521.34 cm^−1^ (C=N stretching vibrations of thiazole), 1256.39 cm^−1^ (C-N stretching vibration of the amine), 1122.36 cm^−1^ (C-O stretching vibrations of tertiary alcohol), and 1039.49 cm^−1^ (S=O stretching vibrations of organic sulfoxide). The results from the FTIR of the CEMNPs showed the existence of prominent peaks of MLX, which indicates there is no interaction occurring between MLX and the polymer during the preparation of nanoparticles.

### 2.2. Encapsulation Efficiency (%)

The encapsulation efficiency (%) as analyzed by HPLC in the supernatant devoid of CEMNPs was found to be 96.49% for the CEMNPs and was in good agreement with Mohammed et al. [23]. The high drug entrapment efficiency of the CEMNPs could be explained by an efficient ionic interaction between MLX, which is a weak acid, with two pKa values (pKa l = 1.09, pKa 2 = 4.18) and chitosan. During the preparation of the CEMNPs, a mixture of MLX and TPP solution was slowly added dropwise to the chitosan solution, which allowed the interaction of the negatively charged MLX and TPP with positively charged amine groups in the chitosan, resulting in the formation of high MLX entrapment [28,48].

### 2.3. In Vitro Release Kinetics

**Figure 6:**
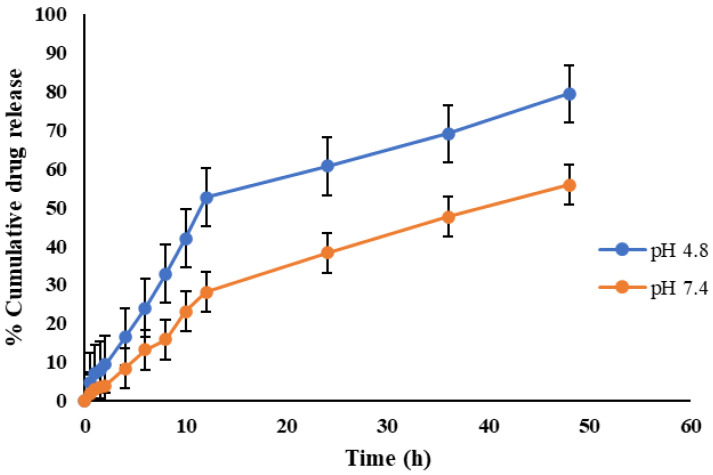
In vitro drug release profile of meloxicam from chitosan-encapsulated meloxicam nanoparticles (CEMNPs) at pH 4.8 and 7.4.

The in vitro cumulative drug release of MLX from the CEMNPs over a period of 48 h at pH 4.8 and 7.4 is depicted in Figure 6. According to the release curves, MLX released from the CEMNPs over a 48 h period was slower at pH 7.4 as compared to pH 4.8. A total amount of 79.36% and 55.86% of loaded MLX was released from the CEMNPs at pH 4.8 and 7.4, respectively, within 48 h. When compared to pH 7.4, the release of MLX from the CEMNPs was more rapid at acidic pH. This might be due to the expanded swelling property of chitosan at acidic pH. Since chitosan is more soluble and degradable in acidic pH than neutral and/or alkaline pH, it is possible that the delayed release of MLX from the CEMNPs at pH 7.4 is caused by the nature of this molecule [49,50]. The controlled release of MLX was observed from the CEMNPs without any initial burst, which infers that the encapsulation of the drug was homogenous. The lack of the initial burst suggests that the drug was properly encapsulated in the nanoparticles, and there was no surface deposition [51,52]. The ability of chitosan to hydrate when in contact with dissolution media results in the formation of gelatinous mass, which acts as a retardant material for the drug to diffuse out, which helps in sustained drug release [53].

The MLX release data from the CEMNPs dispersion was subjected to kinetic analysis using several kinetic models, and the results are presented in Table 1. The kinetic model with the best computed correlation coefficient (*R*^2^) was chosen as the MLX release mechanism. The release data of MLX from the CEMNPs dispersion was better explained by the Baker–Lonsdale models at both pH points, which indicates that the drug release was happening from the matrix type of formulation and nano capsules. According to the release exponent ‘n’, calculated using the Korsmeyer–Peppas equation, the drug’s diffusion from the CEMNPs was anomalous and non-Fickian, with values of 0.66 and 0.67 at pH 4.8 and 7.4, respectively. These results are consistent with the earlier published kinetic investigations of MLX release data from chitosan nanoparticles [28].

### 2.4. In Vivo Pharmacokinetics

**Figure 7:**
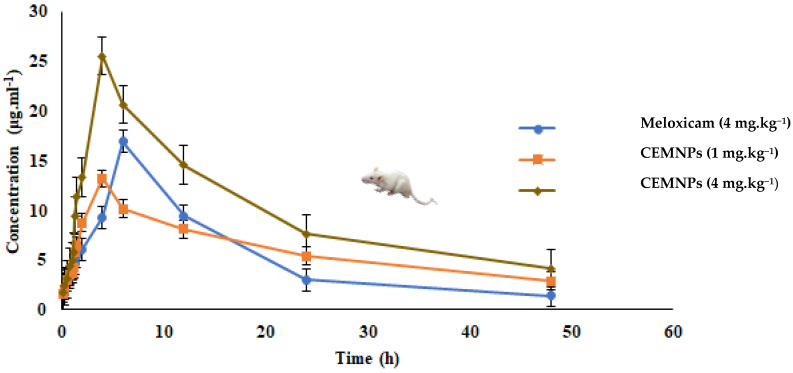
Mean (±SE) plasma concentration vs. time profile of meloxicam following single oral administration of meloxicam (4 mg·kg^−1^) and high (4 mg·kg^−1^) or low (1 mg·kg^−1^) dose of chitosan-encapsulated meloxicam nanoparticles (CEMNPs) in male Wistar rats (n = 3 at each time interval).

The oral pharmacokinetics of meloxicam were evaluated in three groups of rats receiving 4 mg·kg^−1^ of MLX and 1 or 4 mg·kg^−1^ equivalent weight of MLX in CEMNPs, respectively. Here, based on the encapsulation efficiency results, MLX quantified in the CEMNPs and the weight of CEMNPs corresponds to 1 mg (1.04 mg of CEMNPs), and 4 mg (4.15 mg of CEMNPs) of MLX was administered to rats. The non-compartment model presented the best fit to the plasma drug concentration time curves of MLX following single oral administration in male Wistar rats. The mean blood MLX concentrations following single oral administration in different groups are shown in Figure 7. Meloxicam whole-blood concentrations increased reaching a maximal concentration in about 6 h for Group-I and 4 h for Group-II and Group-III. Then, MLX concentration decayed with a half-life of 11.98 ± 0.175 h, 23.416 ± 2.471 h, and 17.130 ± 0.263 h for the doses of 4 mg·kg^−1^ of MLX and 1 or 4 mg·kg^−1^ of CEMNPs, respectively (Figure 7). Relevant pharmacokinetic parameters are shown in Table 2. The half-life, AUC, AUMC, C_max_, MRT, and Vd_ss_ was significantly (*p* < 0.05) differed between MLX and CEMNPs groups.

The mean area under the curve (AUC_0–48_) is considered as an indicator of the extent of absorption and was non-significantly higher (*p* > 0.05) for 1 mg·kg^−1^ CEMNPs (289.749 ± 17.993 μg·mL·h^−1^) and significantly higher (*p* < 0.05) for 4 mg·kg^−1^ CEMNPs group rats (477.552 ± 7.99 μg·mL·h^−1^), in comparison to MLX (256.569 ± 2.022 μg·mL·h^−1^). Thus, the rate and extent of MLX absorption is similar to 1 mg·kg^−1^ CEMNPs group rats and increased at least 2-fold for 4 mg·kg^−1^ CEMNPs group rats than 4 mg·kg^−1^ plain MLX in rats.

The mean apparent volume of the distribution of MLX was 0.265 ± 0.006 L.kg^−1^, 0.088 ± 0.016 and 0.183 ± 0.016 for 4 mg·kg^−1^ of MLX, and 1 or 4 mg·kg^−1^ of CEMNPs, respectively, following oral administration. This indicates that the apparent volume of distribution is substantially higher for plain MLX as compared to CEMNPs. Thus, CEMNPs may have better target efficiency.

The oral clearance (Cl/F_obs) for 1 mg·kg^−1^ of CEMNPs [0.00258198 ± 0.000069 (mg/kg)/(μg/mL)/h] and 4 mg·kg^−1^ of CEMNPs [0.00690 ± 0.0002 (mg/kg)/(μg/mL)/h] were significantly lower (*p* < 0.05) in comparison to 4 mg·kg^−1^ of plain MLX [0.0142 ± 0.00008 (mg/kg)/(μg/mL)/h]-treated rats. This indicates that the oral clearance was significantly decreased for the CEMNPs-treated rats as compared to plain MLX.

The mean area under the plasma MLX concentration-time curve from zero to infinity (AUC_0–∞_), half-life, and mean resident time of 1 mg·kg^−1^ of CEMNPs was 1.4-, 2-, and 1.8-fold greater than 4 mg·kg^−1^ of MLX standard drug. The possible reason for the above-mentioned results is mainly due to the protective effect of the chitosan. This is because the encapsulated MLX needs to be released from the CEMNPs into circulation and then distributed to tissues and eliminated [37].

Meloxicam is an enolic acidic NSAID drug with anti-inflammatory, analgesic, and antipyretic activities [8,9]. The aqueous thermodynamic solubility and dissolution rate of MLX is poor to low. The low molecular weight of 351.4 kDa helps to maintain the well permeability. However, MLX has an 89% bioavailability after dissolution, and its poor dissolving property restricts both its absorption rate and onset of action [17].

In the present study, we have prepared MLX nanoparticles with chitosan to improve absorption rate with less GI side effects. The features of chitosan-based nanomaterials, such as the protection of pharmaceuticals against gastrointestinal degradation, higher mucoadhesion, improved permeability, controlled drug release, and efflux pump suppression, have enhanced the fast and sustained drug delivery [29,30,54,55,56].

Kürti et al. [17] have reported similar kind of results with MLX-polyvinylpyrrolidone nanoparticle pharmacokinetics in male Sprague–Dawley rats. The C_max_ and AUC were increased 2.4- and 2-fold, respectively, when MLX-polyvinylpyrrolidone nanoparticles were administered orally to rats, as compared to plain MLX.

The results of this study were in similar to Nagai et al. [18], who designed and studied the pharmacokinetics of MLX solid nanoparticles in male Dark Agouti rats. The time taken to reach the maximum concentration (T_max_) of MLX solid nanoparticles was shorter than that of plain MLX, and the intestinal penetration of MLX-NPs was significantly higher in comparison with plain MLX. The area under the plasma MLX concentration-time curve for MLX-NPs was 5-fold higher than that for plain MLX, and the AUC in rats administered 0.05 mg/kg MLX-NPs were similar to rats administered the therapeutic dose of 0.2 mg/kg plain MLX.

The prolonged MRT of MLX in the present study was in accordance with the reports of Wang et al. [57], who studied the in vivo pharmacokinetics of hybrid thermosensitive MLX chitosan gel in Sprague–Dawley rats. The MLX chitosan–glycerol gel significantly prolonged the MLX elimination time by 24 h, and a 1.8-fold increase in ACU was observed compared to plain MLX.

## 3. Materials and Methods

### 3.1. Chemicals and Reagents

Meloxicam standard (M/s. Sigma-Aldrich, St. Louis, MO, USA), Meloxicam I.P grade (received as gratis from M/s. Zenex animal health India Pvt. Ltd., Gujarat, India), Chitosan (low molecular weight), and Sodium tripolyphosphate were procured from M/s. Sigma-Aldrich, St. Louis, MO, USA. Acetic acid glacial (M/s. Merck, Kenilworth, NJ, USA), HPLC grade acetonitrile, methanol, and 70% perchloric acid were procured from M/s. Hi-media, India, and carboxymethylcellulose (M/s. SD fine-chem limited, Chennai, India) and HPLC grade water (M/s. Hi-media, Mumbai, India) were procured commercially and used in the study.

### 3.2. Preparation of Chitosan-Encapsulated Meloxicam Nanoparticles (CEMNPs)

Ionic gelation was used for the preparation of chitosan-encapsulated meloxicam nanoparticles (CEMNPs) with minor modification in the method described by Duse et al. [58]. In a nutshell, 1 mg·mL^−1^ of chitosan was dissolved in 0.5% acetic acid solution and stirred continuously at 120 rpm over the course of the night at room temperature. An aqueous solution of sodium tripolyphosphate (NaTPP) at a concentration of 1 mg·mL^−1^ was prepared. Meloxicam was dissolved in methanol at a concentration of 1 mg·mL^−1^ solution, and desired volume was mixed with NaTPP solution. The CEMNPs were prepared by stirring the chitosan solution at 600 rpm using a magnetic stirrer, and dropwise meloxicam-NaTPP solution was added at the ratio of 3:1 (Chitosan:NaTPP). After being brought to a pH of 5.5, the obtained nanoparticle suspension was agitated for 30 min and then centrifuged at 16,000 rpm for 30 min at 14 °C. The supernatant was removed, and the wet pellet of CEMNPs was collected and washed with ascending grade ethanol. Then, they were lyophilized with freeze-dryer (ScanLaf, CoolsafeFreezedryer, LaboGene; LillerØd, Denmark), and the powder form CEMNPs was stored at 4 °C for further analysis.

### 3.3. Characterization of CEMNPs

#### 3.3.1. Transmission Electron Microscopic (TEM) and Scanning Electron Microscopic (SEM) with Energy Dispersive Spectroscopy (EDS) Analysis

The TEM analysis was conducted using JOEL 30,100 TEM machine to determine the size and shape of TiO_2_ NPs. Thin films of the sample were prepared on a TEM grid by just dropping a very small amount of the sample on the grid, extra sample was removed, and then the film on the TEM grid was allowed to dry by putting it under a mercury lamp for 5 min.

The SEM analysis was conducted using CARL-ZEISS AG-ULTRA 55 machine equipped with EDS analyzer to determine the size, shape, and elemental distribution of CEMNPs. Thin films of the sample were prepared on a SEM grid by just dropping a very small amount of the sample on the grid, extra sample was removed, and then the film on the SEM grid was allowed to gold sputter (EMITECHK-550X; Ashford, UK) for 1 min before taking the images and EDS analysis.

#### 3.3.2. Zetasizer Analysis

The dynamic light scattering (DLS) approach with *zeta* sizer (Nanopartica^®^, HORIBA, SZ-100; Kyoto, Japan) was used to assess the mean hydrodynamic diameter, *zeta* potential, and polydispersity index of CEMNPs in carboxymethyl cellulose (CMC; 0.1 *w*/*v*).

The instrument was equipped with 10 mW or 100 mW laser beam at a wavelength of 532 nm. Surface charge of CEMNPs was measured by an electrophoretic light scattering technique using a fold capillary cuvette. *Zeta* potential was measured with an electrode voltage of 3.9 V at 25 °C.

#### 3.3.3. UV-Visible Spectra Analysis

The UV-VIS spectrophotometer (UV-2450, Shimadzu, Kyoto, Japan) was employed to assess the MLX and CEMNPs’ UV-visible spectrum after diluting a tiny amount of the sample in distilled water and scanning for absorption maxima.

#### 3.3.4. Fourier Transform Infrared (FTIR) Spectroscopy

FTIR spectral analysis of MLX and CEMNPs were performed using a FT/IR 6600 FTIR spectrometer (Jasco Corporation, Tokyo, Japan) over a range from 4500 to 350 cm^−1^. Potassium bromide was used for the preparation of respected sample compressed discs. The number of scans used was 16 at a resolution of 4 cm^−1^ with a scanning speed of 2 mm per second. FTIR was used to evaluate the types of functional groups present in the prepared nanoparticles and will confirm the presence of drug after successful encapsulation.

#### 3.3.5. Encapsulation Efficiency

The percent encapsulation efficiency (EE) was evaluated by the method described by Chuah et al. [59] with slight modifications. The prepared CEMNPs were centrifuged at 12,000 rpm for 30 min. The supernatant containing unbound MLX was separated, and the concentration was determined by using a validated reverse phase (C_18_) high-performance liquid chromatography (HPLC; Shimadzu^®^, Japan) assay [54]. The EE% was calculated using the following Equation (1):

(1) $$\mathrm{EE}\%=\frac{\left(\mathrm{Total}\;\mathrm{amount}\;\mathrm{of}\;\mathrm{meloxicam}-\mathrm{amount}\;\mathrm{of}\;\mathrm{free}\;\mathrm{meloxicam}\right)}{\mathrm{Total}\;\mathrm{amount}\;\mathrm{of}\;\mathrm{meloxicam}}\times 100$$

#### 3.3.6. In Vitro Release Kinetic Studies

The drug release kinetics (in vitro) were carried out by using the dialysis membrane as reported by Rajashekaraiah et al. [38], with minor modifications. The dialysis bag technique entails dispersing 3 mg of the CEMNPs in 1 mL of the release medium and placing in a presoaked dialysis bag (12,000–14,000 MWCO, M/s. Sigma-Aldrich, St. Louis, MO, USA, surface area of 20.5 cm^2^), which was then submerged in a beaker that contained 50 mL of the test media maintained at 37 ± 0.5 °C in a temperature-controlled shaking water bath at 50 rpm, maintained for 48 h. The release media were phosphate buffer solution (PBS) of pH 7.4 and acetate buffer of pH 4.8. At predetermined time intervals (0.5, 1.0, 1.5, 2.0, 4.0, 6.0, 8.0, 10.0, 12.0, 24.0, and 48.0 h), 1 mL aliquots were collected and replaced with the same volume of fresh media to maintain sink conditions. The MLX concentration in release media was determined using HPLC [60]. Each sample has triplicate copies of the analytical run. The intensity of absorption was plotted against time, which gave the desorption profile of CEMNPs. The cumulative percent release was calculated for each time point. The data obtained was subjected to windows-driven KineticDS3 software [61] to get the best-fitted in vitro release kinetic model to determine the rate of MLX release from CEMNPs. Different mathematical modeling drug release equations were applied to perform drug release kinetics such as zero-order Equation (2), first-order Equation (3), Korsmeyer–Peppas Equation (4), Higuchi Equation (5), and Baker–Lonsdale Equation (6):

*Q_t_* = *Q*_0_ − *K*_0_*t* (2)

where *Q_t_* is the cumulative amount of drug release, *Q*_0_ is the initial amount of drug, *K*_0_ is the zero-order release constant, and *t* is the time.

*log C* = *log C*_0_ − *K_t_*/2.303 (3)

where *C*_0_ is the initial concentration of drug, *K* is the first order rate constant, and *t* is the time.

*M_t_*/*M* = *K^tn^* (4)

where *M_t_/M* is the cumulative drug release, *K* is the release constant, *t* is the time, and *n* is the release exponent.

*Q* = *Kt*^1/2^ (5)

where *Q* is the cumulative drug release, *K* is Higuchi release constant, and *t* is the time.

*f* = 3/2 [1 − (1 − *Q*)^2/3^] − *Q* = *Kt* (6)

where *K* is the rate constant, and *t* is the time.

### 3.4. Meloxicam Determination

#### 3.4.1. Instrumentation

The HPLC system (Shimadzu^®^, Japan) consisted of an LC-20AD quaternary gradient pump, a rheodyne manual injector with 20 µL loop, and an SPD-20 AV UV-Vis detector. The analytical column employed was reverse phase (RP-C_18_) column (4.6 mm × 250 mm, 5 µm particle size). The mobile phase comprised 46 parts of solution ‘A’: acetonitrile and 54 parts of solution ‘B’: 1% glacial acetic acid, in the ratio of 46:54 (*v*/*v*). The mobile phase liquid was filtered through membrane filter (0.22 µm diameter) and later degassed with the help of ultrasonic cleaner (Sonica^®^, Soltec Soluzion Technologiche, Basaldella, Italy). The flow rate was kept at 1.0 mL·min^−1^ with run time of 10 min for each sample. Chromatography was performed at 40 °C with detection at 355 nm using UV detector [60]. The room temperature was maintained at 23 °C during the assay. The concentration of the analyte in the samples was determined from the standard curve constructed for the purpose. The retention time of MLX in the present assay study was 7.248 min.

#### 3.4.2. Standardization and Validation of Assay

A stock solution of MLX standard with a concentration of 100 µg·mL^−1^ was prepared by dissolving 5 mg of MLX standard in 50 mL of methanol. Working stock solutions with concentrations of 0, 0.25, 1, 5, 10, 15, 25, 50, and 100 µg·mL^−1^ were created by diluting the MLX stock standard in methanol. In order to avoid cross-contamination and evaporation, standards were aliquoted into 2 mL amber-colored vials wrapped with foil to block light and stored at −20 °C.

**Figure 8:**
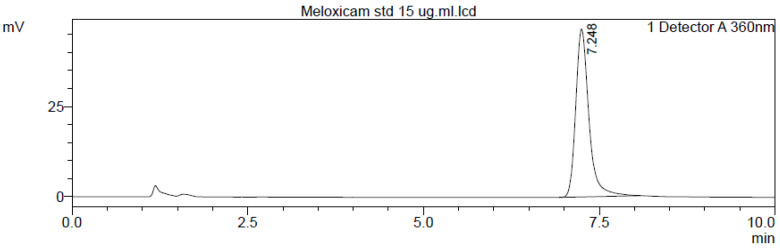
Chromatogram for a plasma spiked with 15 g·mL^−1^ of standard meloxicam.

**Figure 9:**
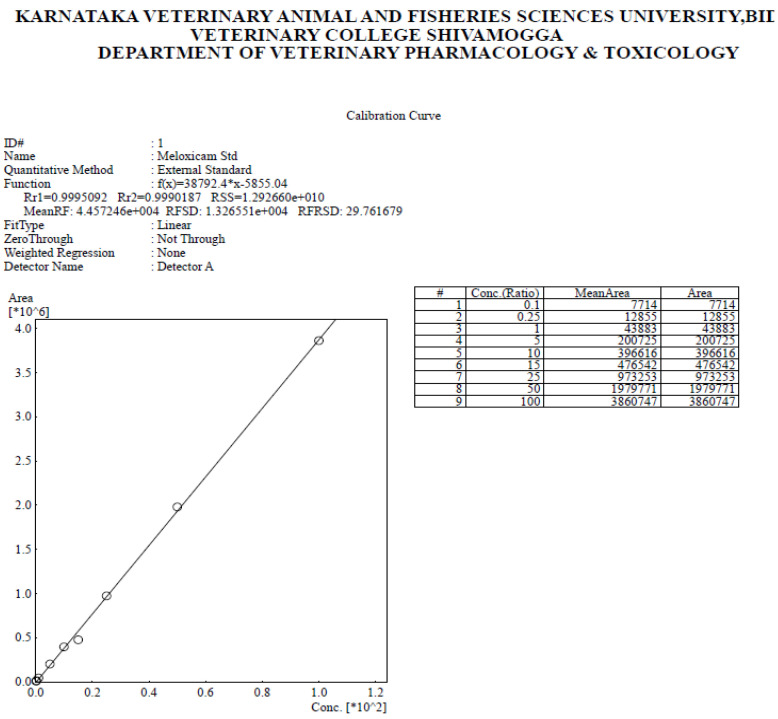
The calibration curve of meloxicam as assessed by HPLC. #—Serial number.

The drug concentration (*X*-axis) was plotted against the peak area (*Y*-axis) to create the standard curve. The standard curve had an *R*^2^ value of 0.999, and it was linear in the range of 0.1 to 100 µg·mL^−1^. A typical MLX chromatogram and calibration curve were depicted in Figure 8 and Figure 9, respectively. The limit of detection (LOD) and limit of quantification (LOQ) were determined to be 2.8 and 8.5 ng·mL^−1^, respectively. The precision and accuracy of the assay were assessed using samples at concentration 1, 10, and 25 μg·mL^−1^. The intraday and interday assay coefficient of variation was less than 7.64% (Table 3), and the percent recovery of MLX in rat plasma was 97.24%.

### 3.5. Pharmacokinetic Studies following Single Oral (p.o) Administration

#### 3.5.1. Experimental Animals

Male Wistar rats (N = 99) of 8 weeks of age and weighing 175–185 g were procured from the authorized vendor (Biogen^®^ Laboratory Animal Facility, Bengaluru-562107, KS, India). MLX is metabolized in the liver through cytochrome P450 2C isoenzymes, particularly CYP450 2C11 isoenzyme, which is absent in female rats. This leads to slower elimination of MLX from female rats. In light of these constraints for female rats, male rats were used for this study [62]. All the rats were housed in Small Animal Facility of Veterinary College, Bengaluru, in polypropylene cages at an ambient temperature of 23 ± 2 °C and 55 ± 5% of relative humidity, with 12 h:12 h of light and dark cycle. Animals were kept on ad libitum feed and water. After one week of acclimatization, the experimental rats were randomly divided into three groups (n = 33). Freshly prepared drug solutions were suspended in carboxymethyl cellulose (CMC) (0.1% *w*/*v*) and subjected to ultrasonication by using an ultrasonic cleaner for 30 min and stirred over vortex agitator for about 5 min in order to obtain homogenized suspension. After overnight fasting, animals were weighed before dosing, and MLX suspension (CMC; 0.1 *w*/*v*) was administered through oral gavage as single dose of 4 mg·kg^−1^ to Group I, CEMNPs @ dose rate equivalent to 1 mg·kg^−1^ of MLX to Group-II, and CEMNPs @ dose rate equivalent to 4 mg·kg^−1^ of MLX to Group-III. Institutional animal ethics committee approval was obtained (VCH/IAEC/2021/123, dated 27 July 2021) prior to experimental studies in Wistar rats, and all maneuvers involved in animal studies were according to the guidelines of Committee for the Purpose of Control and Supervision of Experiments in Animals (CPCSEA, New Delhi, India).

#### 3.5.2. Blood Sampling

Blood samples were collected at 0.0, 0.16, 0.25, 0.5, 0.75, 1.0, 1.25, 1.5, 1.75, 2.0, 4.0, 6.0, 12.0, 24.0, and 48.0 h postdosing from experimental rats and transferred to heparinized tube. Multiple numbers of rats were used for serial collection of blood in some time points, with at least 18 h intervals between collections. The blood samples were centrifuged at 5500 rpm, and the resultant plasma was stored at −80 °C until subjected to quantitative analysis of MLX.

#### 3.5.3. Plasma Samples for HPLC Analysis

To 0.1 mL of plasma in 2 mL Eppendorf tubes, 0.55 mL of a 0.1 M sodium dihydrogen phosphate buffer (pH 3.3) was added. Proteins were precipitated by the addition of 50 µL of 70% perchloric acid, and tubes were agitated in a vortex mixer for minimum of one min. To this solution, 50 µL of acetonitrile was added, and the tubes were agitated again for one min. The final solution was centrifuged for 10 min at 4500 rpm. Following centrifugation, a 0.22 µm nylon membrane syringe filter was used to separate the clear supernatant, and 20 µL of this was injected into HPLC system for quantifying MLX [22,60].

#### 3.5.4. Pharmacokinetic (PK) Analysis of Meloxicam

The mean plasma MLX concentration vs. time curve was plotted for all the three groups. The pharmacokinetic parameters were derived by using a menu-driven add-in program for Microsoft Excel written in visual basic (PK Solver; version 2.0) validated previously [63]. After subjecting the data of mean plasma MLX concentration vs. time intervals of different groups to a non-compartment model with no lag time, the pharmacokinetic variables were derived.

### 3.6. Statistical Analysis

The mean plasma MLX concentration and pharmacokinetic data obtained were subjected to one-way ANOVA. Tukey’s post hoc multiple comparison test using GraphPad Prism software program (GraphPad^®^ software Inc., Version 8.4.3; San Diego, CA, USA) was applied to determine the significant difference between the set of data. The difference was considered significant at *p* < 0.05 or lower.

## 4. Conclusions

In conclusion, compared to plain MLX, the CEMNPs have rapid absorption through oral administration, a lengthy half-life, long mean residence time, and comparatively low apparent volume of distribution, and clearance demonstrated the sustained releasing impact of CEMNPs. This pharmacokinetic trait contributed to the explanation of the enhanced drug delivery, rapid onset of action, and extended activity of MLX following a single low oral dose of chitosan-encapsulated meloxicam nanoparticles in experimental rats. This chitosan-based drug-delivery system appears to be promising for sustained drug delivery based on the correlation between the in vitro and in vivo outcomes of this study and thus necessitates the appropriate clinical studies for potential application in therapeutics.

## Figures and Tables

**Table 1:** Kinetic analysis of the in vitro release data of meloxicam from chitosan-encapsulated meloxicam nanoparticles.

	pH	*n*	*R* ^2^				
			Korsmeyer–Peppas Model	Zero Order	First Order	Higuchi	Baker–Lonsdale
CEMNPs	4.0	0.66	0.994	0.858	0.11	0.276	0.982
	7.4	0.67	0.997	0.939	0.127	0.217	0.988

**Table 2:** Pharmacokinetic variables of following single oral administration of meloxicam (4 mg·kg^−1^) and high (4 mg·kg^−1^) or low (1 mg·kg^−1^) dose of chitosan-encapsulated meloxicam nanoparticles (CEMNPs) in male Wistar rats (n = 3 at each time interval).

Kinetic Parameter	Unit	Mean ± SE		
		Group-1	Group-2	Group-3
λ_z_	1/h	0.0578 ± 0.0008	0.0296 ± 0.002	0.0404 ± 0.0005
t_1/2_	h	11.980 ± 0.175 ^a^	23.416 ± 2.471 ^b^	17.130 ± 0.263 ^a^
T_max(obs.)_	h	6	4	4
C_max(obs.)_	μg·mL^−1^	16.905 ± 0.267 ^a^	13.185 ± 1.366 ^b^	25.52 ± 0.174 ^c^
AUC_(0–48)_	μg/mL·h	256.569 ± 2.022 ^a^	289.749 ± 17.993 ^a^	477.552 ± 7.99 ^b^
AUC_(0–∞)_	μg/mL·h	280.875 ± 1.60	387.299 ± 10.424	579.592 ± 16.596
AUMC_0-inf(obs.)_	μg/mL·h^2^	5230.04 ± 67.82 ^a^	13230.251 ± 961.288 ^b^	15395.995 ± 921.371 ^b^
MRT_0-inf(obs.)_	h	18.62 ± 0.339 ^a^	34.160 ± 3.000 ^b^	26.563 ± 0.832 ^c^
Vz/F_(obs.)_	(mg/kg)/(μg/mL)	0.246 ± 0.004	0.0872 ± 0.011	0.170 ± 0.002
Cl/F_(obs.)_	(mg/kg)/(μg/mL)/h	0.0142 ± 0.00008	0.00258198 ± 0.000069	0.00690 ± 0.0002
Vd_ss_	L·kg^−1^	0.265 ± 0.006 ^a^	0.088 ± 0.016 ^b^	0.183 ± 0.0005 ^c^

Note: Group-I rats received 4 mg·kg^−1^ of MLX; Group-II rats received 1 mg·kg^−1^ of CEMNPs; Group-III rats received 4 mg·kg^−1^ of CEMNPs; λ_z_ = Terminal rate constant; t_1/2_ = half-life; T_max(obs.)_ = observed time to reach maximum concentration; C_max(obs.)_ = observed maximum concentration; AUC_(0–48)_ = the area under the meloxicam concentration-time curve from time ‘0’ h to 48 h; AUC_(0–∞)_ = the area under the meloxicam concentration-time curve from time ‘0’ h to ‘∞’; AUMC_0-inf_ = the area under the first moment of the meloxicam concentration-time curve from time ‘0’ h to ‘∞’; MRT= mean residence time; Vd_ss_ = steady state volume of distribution derived as Dose (mg·kg^−1^) × AUMC/(AUC)^2^; C_last(obs.)_ = last observed concentration. Values bearing dissimilar small alphabets with in a column vary significantly at *p* < 0.05.

**Table 3:** Intraday and Interday assay coefficient of variance (% CV) in assay of meloxicam using HPLC.

Concentration in Plasma (µg·mL^−1^)	Intraday Assay CV (%)	Interday Assay CV (%)
1	7.64	5.94
10	5.12	7.15
25	3.15	6.49

## Data Availability

The data presented in this study are available on request from the corresponding author.

## Supplementary Materials

The following supporting information can be downloaded at: https://www.mdpi.com/article/10.3390/molecules27217312/s1, Table S1: Elemental distribution obtained from EDS analysis of CEMNPs.

## Sample Availability

Samples of the compound are available from the authors.
