# Supplementary material for: Chitosan Encapsulated Meloxicam Nanoparticles for Sustained Drug Delivery Applications: Preparation, Characterization, and Pharmacokinetics in Wistar Rats
Source: Molecules. 2022 Oct 27;27(21):7312. doi: 10.3390/molecules27217312 (PMC9658985; doi:10.3390/molecules27217312)
Supplement: Supplementary file 1 [file molecules-27-07312-s001.zip › molecules-1913647-supplementary.pdf]

molecules

MDPI

Supplementary Table S1. Elemental distribution obtained from EDS analysis of CEMNPs.

Element	Weight%	Atomic%
C K	68.10	76.19
N K	10.19	9.78
O K	11.73	9.85
S K	9.99	4.19
Totals	100.00	
